# Distinct cytokine-producing dendritic cell profiles in females and males with major depressive disorder

**DOI:** 10.3389/fncel.2026.1753241

**Published:** 2026-02-09

**Authors:** Anna-Lena Boller, Jana Freff, Maximilian Bast, Kathrin Schwarte, Udo Dannlowski, Bernhard T. Baune, Stefanie Scheu, Judith Alferink

**Affiliations:** 1 Department of Psychiatry, University of Münster, Münster, Germany; 2 Cells in Motion Interfaculty Cluster, University of Münster, Münster, Germany; 3 Institute for Translational Psychiatry, University of Münster, Münster, Germany; 4 Department of Psychiatry, Medical School and University Medical Center OWL, Protestant Hospital of the Bethel Foundation, Bielefeld University, Bielefeld, Germany; 5 Department of Psychiatry, University of Melbourne, Melbourne, VIC, Australia; 6 The Florey Institute of Neuroscience and Mental Health, University of Melbourne, Melbourne, VIC, Australia; 7 Institute of Immunology, University Medical Center Rostock, Rostock, Germany; 8 Institute of Medical Microbiology and Hospital Hygiene, Medical Faculty and University Hospital Düsseldorf, Heinrich Heine University, Düsseldorf, Germany

**Keywords:** cytokines, dendritic cells, female, IL-23, immune system, inflammation, major depressive disorder, male

## Abstract

**Introduction:**

Major depressive disorder (MDD) is a revalent and disabling condition increasingly associated with immune dysregulation. Dendritic cells (DCs) are key immune sentinels that shape inflammatory responses and T-cell polarization, including Th17 pathways implicated in depression-related mild inflammation. Given well-documented sex differences in immune responses and cytokine profiles in MDD, differential DC activation may represent a mechanistic link between sex-associated immune cell profiles in depression. However, DCs remain insufficiently characterized in MDD.

**Methods:**

We performed an exploratory study using flow cytometry-based immunophenotyping to assess circulating DC subsets, including CD1c^+^ and CD141^+^ conventional DCs (cDCs), plasmacytoid DCs (pDCs), and their cytokine profiles in individuals with MDD (*n* = 55) and healthy controls (HC, *n* = 32). Stratification by depression severity and sex, together with correlation and multivariate linear regression analyses, and cluster analysis, was used to examine associations between DC subsets and depressive symptom severity in females and males.

**Results:**

Stratification by HAM-D17 scores revealed reduced counts of pDCs and increased frequencies of CD11c^+^ cDCs in the peripheral blood (PB) of severely depressed participants compared to HC or mildly depressed patients, respectively. Regarding cytokine-producing DCs, sex-stratified analyses showed that frequencies of IL-23^+^ cDCs were elevated and symptom-associated only in females with MDD compared to sex-matched controls, whereas frequencies of TNF^+^, IL-1β^+^, and IL-6^+^ cDCs were selectively increased in depressed males. Cluster analyses identified distinct female- and male-specific DC subset patterns distinguishing individuals with MDD from HC. Multivariate linear regression revealed a significant predictive contribution of cytokine-producing DCs, together with age and BMI, in females but not in males.

**Discussion:**

These findings demonstrate sex-specific alterations in cytokine-producing DCs in MDD and a strong association between IL-23^+^ cDCs and depressive symptom severity, suggesting a key role for these cells in immune dysregulation, particularly in females with depression.

## 1 Introduction

Major depressive disorder (MDD) is a common mental illness and one of the leading causes of disability worldwide (McIntyre and Lee, 2016; Otte et al., 2016). At least one-third of patients experience recurrent episodes, contributing to its chronic and relapsing course (Kessler and Bromet, 2013). The etiology of MDD involves a complex interplay of genetic and environmental factors, including common and rare genetic variants, as well as environmental stressors (Otte et al., 2016; Carvalho Silva et al., 2024; Kendall et al., 2021; Krishnan and Nestler, 2008; Schramm et al., 2020). Beyond these well-established contributors, growing evidence points to immune dysregulation as a key pathophysiological mechanism in MDD. Elevated levels of pro-inflammatory cytokines and chemokines, together with altered frequencies and increased activation of immune cells in peripheral blood (PB), have been reported in individuals with depression (Beurel et al., 2020; Miller, 2025; Miller et al., 2009). Within this inflammatory context, dendritic cells (DCs) are of particular interest due to their central role in linking innate and adaptive immune responses (Guilliams et al., 2016; Steinman, 2012). However, their cytokine-producing capacity and relationship with depressive symptoms and severity have not yet been investigated.

DCs are a heterogeneous group of peripheral immune cells specialized in antigen presentation (Segura, 2016). They recognize pathogen-associated (PAMPs) and damage-associated molecular patterns (DAMPs) that signal infection or cellular stress, thereby initiating adaptive immune responses by presenting antigenic peptides to naïve T cells (Guilliams et al., 2016; Mildner and Jung, 2014). Upon antigen uptake within a specific cytokine milieu, DCs upregulate MHC class II, costimulatory molecules, and chemokine receptors, enabling migration to lymph nodes to prime naïve T cells (Segura and Villadangos, 2009). In humans, conventional DCs (cDCs) comprise CD1c^+^ cDCs (DC2), the most abundant subset in PB, which primarily activate CD4^+^ T cells, and CD141^+^ cDCs (DC1), a smaller subset specialized in cross-presentation to CD8^+^ T cells and antiviral or antitumor immunity (Balan et al., 2019; Collin and Ginhoux, 2019). Furthermore, type 3 DCs (DC3s) were discovered in humans and mice, as an independent lineage of DCs arising from Ly6C^+^ monocyte-DC progenitors. DC3 have been attributed a high capacity for the *in vitro* induction of Th17 cells (Liu et al., 2023; Villani et al., 2017).

DCs produce a broad range of cytokines, including TNF, IL-1β, IL-6, IL-10, IL-12, and IL-23, many of which have been reported to be elevated in the PB of individuals with depression (Collin and Bigley, 2018; Farooq et al., 2017; Haapakoski et al., 2015; Kohler et al., 2017; Leite Dantas et al., 2021). Additionally, DCs exert anti-inflammatory functions by secreting IL-10 and transforming growth factor (TGF)-*β*, promoting regulatory T-cell (Treg) expansion, and upregulating indoleamine 2,3-dioxygenase (IDO), a key enzyme in tryptophan metabolism implicated in MDD (Leite Dantas et al., 2021; Mellor and Munn, 2004). Plasmacytoid DCs (pDCs) represent a minor subset of circulating DCs, and a small subset of those cells produces high amounts of type I interferons (IFNs) during antiviral responses (Ali et al., 2019; Reizis, 2019; Mann-Nuttel et al., 2021).

Sex has a significant impact on immune function, with males and females differing in specific innate and adaptive immune responses, which may contribute to their differential susceptibility to autoimmune diseases and viral infections (Klein and Flanagan, 2016). Growing evidence indicates that sex-specific biological mechanisms influence the vulnerability, course, and treatment outcomes of depression. Women show a higher prevalence of MDD and experience greater rates of residual symptoms and treatment resistance, potentially reflecting differences in genetic regulation of neural circuitry and plasticity, neurotransmitter systems, hormonal milieu, and immune modulation (Di Benedetto et al., 2024; Kuehner, 2003). Supporting this, a recent meta-analysis of inflammatory profiles in adults with MDD reported that women with depression exhibited significantly higher circulating levels of CRP and IL-6 compared to healthy women, whereas these associations were absent in men (Jarkas et al., 2024). These findings underscore the importance of accounting for biological sex in studies investigating cytokine production in depression.

Few studies have examined DCs in humans or rodent models of depressive disorder. A recent human study using immunophenotyping of circulating immune cells in MDD found that effector memory CD4^+^ T cells negatively predicted improvement of depressive symptoms after 3 months of treatment, whereas pDCs predicted a reduction in hopelessness (Stolfi et al., 2025). Another human study identified DCs as mediators linking social stress to reduced efficacy of immunogenic chemotherapy (Yang et al., 2019). This effect was driven by glucocorticoid-induced upregulation of TSC22D3 (GILZ), which impairs DC antigen presentation (Cohen et al., 2006; Vetillard and Schlecht-Louf, 2018). Elevated TSC22D3 expression correlated with higher cortisol, negative mood, and poor treatment response, while in mice, stress-induced glucocorticoid signaling increased TSC22D3 in tumor-infiltrating DCs suppressing type I IFN and T cell-derived IFN-*γ* responses (Yang et al., 2019). Depression-associated behaviors have also been shown to alter DC phenotype and function. Six days of social disruption stress induced a mature, pro-inflammatory profile in splenic DCs of subordinate mice, characterized by increased MHC class I, CD80, CD44, TNF, and IL-6 expression and glucocorticoid resistance (Powell et al., 2009). Adoptive transfer of these DCs enhanced antiviral T-cell responses and tumor immunity in naïve recipients (Powell et al., 2011). Our previous work demonstrated stress-induced alterations in DC phenotype and cytokine profiles in a mouse model of depression-like behavior. We found that chronic social defeat stress reduced splenic DC frequencies, while stress-susceptible mice exhibited enhanced cDC maturation characterized by elevated MHC class II and CD80 expression, increased Th17 cells, and higher corticosterone levels (Leite Dantas et al., 2021; Ambree et al., 2018).

In the present study, we characterized circulating DCs in individuals with MDD and HC, stratified by depression severity and sex, and examined their associations with depressive symptoms and their predictive value for disease severity. Our findings reveal alterations in DC populations and their effector cytokines in MDD, suggesting a contributory role in the immunopathophysiology of depression.

## 2 Materials and methods

### 2.1 Patients and biomaterial

The Department of Mental Health at the University Hospital Münster, Münster, Germany, recruited patients with major depressive disorder (MDD, *n* = 55) and healthy controls (HC, *n* = 32). This study was approved by the Ethics Committee of the Medical Faculty of the University of Münster (#2009-019-f-S) and performed in accordance with the Declaration of Helsinki. All participants provided written informed consent prior to the study. Due to technical reasons, 9 blood samples could not be analyzed for DC subsets, 2 samples could not be included in the cytokine analysis for IL-6 and IL-10. MDD patients were admitted for inpatient psychiatric treatment, and MDD diagnosis was confirmed by two trained psychiatrists according to ICD-10 guidelines. The clinician-rated Hamilton Rating Scale of Depression (HAM-D17) was used to assess severity and only patients with a diagnosis of MDD and a HAM-D17 score ≥ 8 were included. Exclusion criteria comprised autoimmune diseases including type I diabetes mellitus, neurological disorders, severe cardiovascular disorders (CVDs), neuroinflammation or other immune-related disorders. Participants with mild, well-controlled hypertension were eligible for inclusion. Inclusion criteria for sex and age matched HC were required to have no history of psychiatric disorders based on DSM-5 and ICD-10 criteria. The same exclusion criteria applied for MDD patients. In addition to the HAM-D17, the Inventory of Depressive Symptomatology - Clinician Rated (IDS-C; *n* = 84), and the Beck’s Depression Inventory-II (BDI-II; *n* = 83), the latter a self-reported questionnaire for depression severity, was administered. To investigate sex-associated differences in the DC profile in this group, patients and HC were stratified into female (HC = 22, MDD = 32) and male (HC = 10, MDD = 23) participants, based on self-disclosure of the participants. A representative calculation of the effect size was performed for the analysis of the IL-23^+^ cDC frequency, comparing females with MDD and HC based on the Mann–Whitney U test. Effect size was calculated as r, with the corresponding Cohen’s d reported for reference (*r* = 0.31; d ≈ 0.62), indicating a medium effect detectable with the current sample sizes. The female HC and MDD groups were matched for age, smoking, and BMI, as were the male HC and MDD groups. In addition, female and male participants were matched within the HC group as well as within the MDD group. Regarding MDD severity, female and male patients were also matched measured by HAM-D17, IDS-C, and BDI-II (Supplementary Table 1). In both female groups (HC and MDD), approximately 55% of participants were either menopausal or postmenopausal. At inclusion, patients received pharmacological treatment including antidepressants (*n* = 53; 96%; female/male: 32/21), antipsychotics (*n* = 38; 69%; female/male: 23/15), mood stabilizers (*n* = 4; 7%; female/male: 1/3), benzodiazepines (*n* = 19; 35%; female/male: 11/8), and/or anxiolytics (*n* = 26; 47%; female/male: 19/7). More detailed information on medication can be found in the Supplementary Table 2. In a subset of this cohort, we previously analyzed PB B and T lymphocyte subsets (Ahmetspahic et al., 2018; Freff et al., 2022).

### 2.2 Blood sample preparation and flow cytometry

Peripheral blood samples were collected from all participants at 8 a.m. Freshly drawn peripheral venous blood (15 mL) was obtained in sterile sodium heparin-treated VACUETTE® tubes (Greiner Bio-One GmbH, Frickenhausen, Germany). Blood sample preparation for flow cytometry was performed as described before (Ahmetspahic et al., 2018; Freff et al., 2022). In brief, to identify DC subsets, whole blood samples were stained using anti-human monoclonal antibodies (mAbs) against CD3/CD14/CD19 (PerCP), CD11c (BV510), CD303 (FITC), CD141 (PE), and CD1c (BV421). Blood sample staining with respective mAbs (Biolegend®, San Diego, CA) was conducted by incubation of biosamples for 45 min at room temperature (RT) with the indicated antibodies protected from light. Afterwards, samples were treated with RBC lysis/fixation solution (Vienna, AUT: eBioscience™) for 15 min at RT. Thereafter, cells were washed with PBS twice and resuspended in FACS buffer (1x PBS, 2% FCS, 0.01% NaN3). Acquiration was performed on a BD FACS CantoTM II flow cytometer (BD Biosciences) and analyzed with the FlowJoTM software v10 (Ashland, OR: Becton®, Dickinson and Company; 2019). DC cell subpopulations were characterized as shown in the gating strategy (Supplementary Figure 1). cDCs were gated on lineage negative cells (CD3^−^/CD14^−^/CD19^−^) and HLA-DR^+^ CD11c^+^ expression and further on termed cDCs, while pDCs were gated on lineage negative cells (CD3^−^/CD14^−^/CD19^−^) and HLA-DR^+^ CD303^+^ expression. To calculate the cell count, the total number of events in the respective gate was divided by the analyzed sample volume and converted to cell count per milliliter (count/ml).

### 2.3 Stimulation of cells and intracellular cytokine staining

Whole blood samples were also stained using three immunophenotyping antibody panels for identification of surface markers and intracellular cytokine production in PB DCs. Therefore, samples (300 μL) were stimulated with 300 μL of X-Vivo15 with lipopolysaccharide (LPS, Sigma, 100 ng/mL), brefeldin A (1:1000, Biolegend®, San Diego, CA), and monensin (1:1000, Biolegend®, San Diego, CA) for 12 h (37 °C, 5% CO2). Thereafter, cell surface marker staining was performed using anti-CD11c and anti-CD303 mAbs by incubation for 45 min at RT protected from light. Afterwards RBC lysis was performed for 15 min at RT, washed with cold PBS twice and cells were then fixed with 1x FIX/PERM solution (eBioscienceTM FoxP3/Transcription Factor Staining Buffer Set, Thermo Fisher Scientific, Waltham, MA) for 45 min, protected from light, at 4 °C. Afterwards, cells were washed twice and stained with anti-human mAbs against TNF (Alexa647), IL-1β (Alexa647), IL-6 (APC), IL-10 (PE), IL-12 (PE), and IL-23 (PE; Biolegend®, San Diego, CA), incubated for 30 min at RT, protected from light. After washing, cells were resuspended in FACS buffer (1x PBS, 2% FCS, 0.01% NaN3) and acquired on a BD FACS CantoTM II flow cytometer and analyzed with the FlowJoTM software v10 (Becton, Dickinson and Company; 2019, Ashland, OR).

### 2.4 Statistical analysis

Obtained immunological data were analyzed using IBM SPSS version 29, and visualization was performed using GraphPad Prism version 9. To evaluate group differences in demographic data, Chi-square tests were performed for categorical variables, and continuous variables were analyzed using t-tests or Mann–Whitney U tests depending on normal distribution. Immunological outcomes were analyzed using t-tests or Mann–Whitney U tests for two-group comparisons, depending on normal distribution. Normally distributed multiple groups were analyzed with One-way analysis of variance (ANOVA) and Tukey’s *post-hoc* analysis, or Welch’s ANOVA with Games-Howell post-hoc analysis, if homogeneity of variance was violated. Non-normally distributed groups were analyzed with Kruskal-Wallis tests with Bonferroni-corrected post hoc analysis. Correlational analysis was performed to analyze the association between immunological parameters and clinical symptoms measured by HAM-D17, IDS-C or BDI-II. To correct for multiple comparisons, the Benjamini-Hochberg procedure was used. Multivariate linear regression (MLR) was performed to evaluate the influence of co-variates and immune parameters on affective/cognitive symptoms and sum score, as assessed by IDS-C. Least Squares Discriminant Analysis (PLS-DA) was used to evaluate how well immune cell frequencies separate HC and patients with MDD in the respective sex group. PLS-DA was conducted using the R package ‘mixOmics’ (v6.24.0). Relative differences in the immune cell frequencies in all four groups were visualized as a heatmap with the R package ‘pheatmap’ (v1.0.13). Statistical significance was set at *p < 0.05; **p < 0.01; ***p < 0.001.

## 3 Results

### 3.1 Sample description

To characterize DC subsets and their cytokine secretion capacity in depression, we recruited individuals diagnosed with MDD (*n* = 55) and healthy controls (HC, *n* = 32). Sample characteristics and group comparisons, including relevant test statistics, are summarized in Table 1. There were no significant differences between groups in sex, age, or smoking status. However, individuals with MDD had a higher body mass index (BMI: 27.11 ± 4.18) compared to the HC group (25.06 ± 4.26, *p* < 0.05). As expected, depression severity was markedly elevated in the MDD group across the clinical rating scales. Mean total scores were significantly higher in patients with MDD than in HC for the Hamilton Depression Rating Scale-17 (HAM-D17: 17.18 ± 4.32 vs. 0.50 ± 0.80, *p* < 0.001), the Inventory of Depressive Symptomatology - Clinician Rated (IDS-C: 33.16 ± 8.46 vs. 1.39 ± 1.56, *p* < 0.001), and the self-reported Beck Depression Inventory-II (BDI-II: 28.59 ± 10.42 vs. 2.53 ± 2.64, *p* < 0.001). Medical treatment of patients is described in Supplementary Table 2.

**Table 1:** Demographics and clinical characteristics of the study sample.

	HC (*n* = 32)		MDD (*n* = 55)		*p*-value
Sex (female/male)	22/10		32/23		0.327
Smoking	7		16		0.432
	HC		MDD		*p*-value
	*M*	*SD*	*M*	*SD*	
Age	53.84	± 13.25	54.96	± 11.01	0.672
BMI	25.06	± 4.26	27.11	± 4.18	< 0.05*
IDS-C	1.39	± 1.56	33.16	± 8.46	< 0.001***
BDI-II	2.53	± 2.64	28.59	± 10.42	< 0.001***
HAM-D17	0.50	± 0.80	17.18	± 4.32	< 0.001***

HC, healthy controls; MDD, major depressive disorder; BMI, body mass index; IDS-C, Inventory of Depressive Symptomatology–clinical interview; BDI-II, Beck Depression Inventory-II; HAM-D17, Hamilton Rating Scale for Depression. Significant effects are in bold print. *p < 0.05; ***p < 0.001.

### 3.2 Reduced counts of circulating pDCs and enhanced percentages of cDCs in MDD

**Figure 1:**
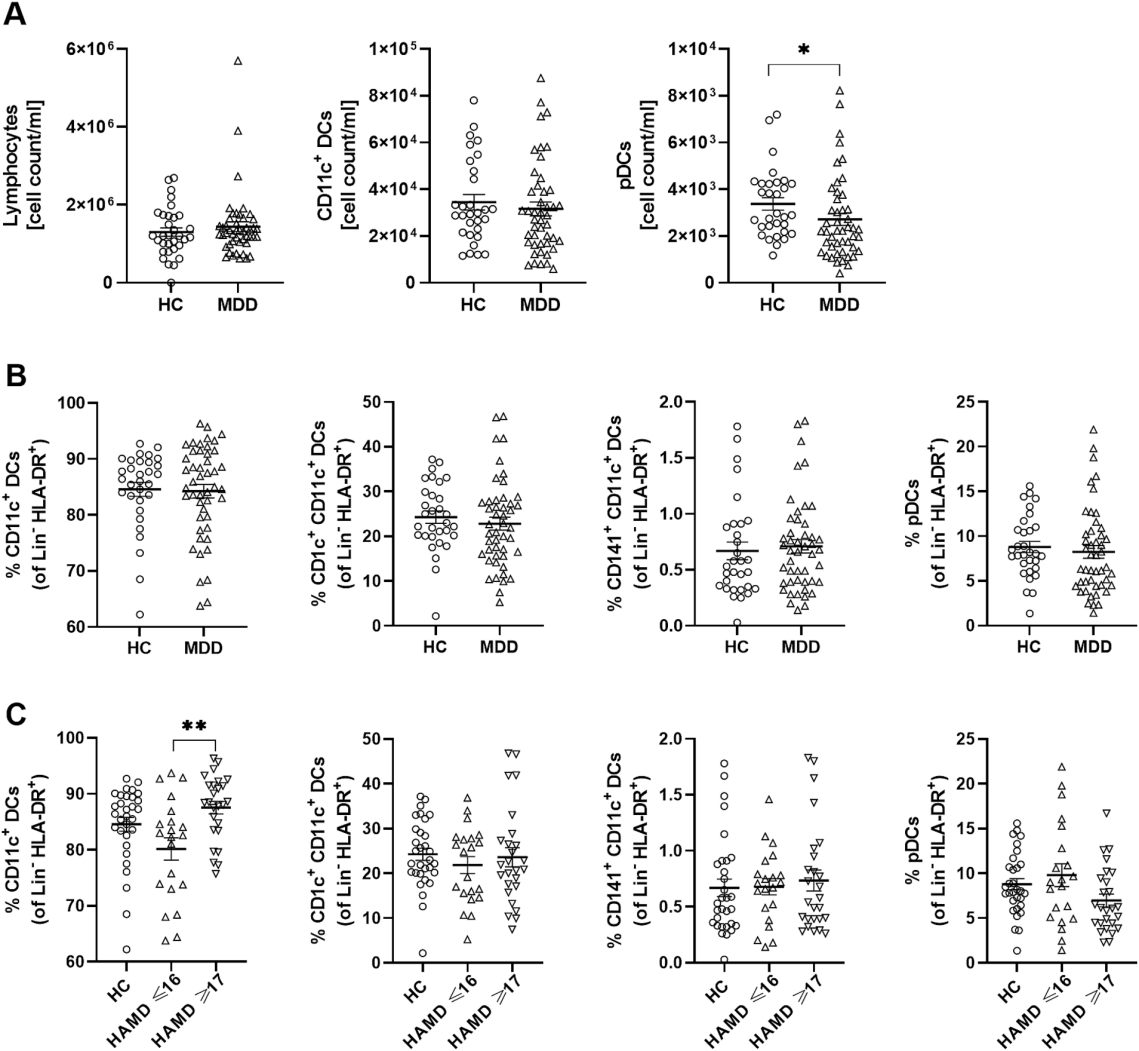
Elevated frequencies of CD11c^+^ cDCs in severely depressed patients. Graphs show cell count/ml in lymphocytes and DC subsets in PB **(A)**, frequencies of DC subsets in healthy controls (HC) and patients with major depressive disorder (MDD) **(B)**. Diagram shows frequencies of DC subsets in participants stratified into HC, mildly depressed (HAMD ≤16) and moderate to severely depressed patients (HAMD ≥17), measured by the Hamilton Rating Scale for Depression (HAM-D17) **(C)**. Cell count/ml and frequencies were determined by multiparameter flow cytometry. Error bars indicate mean ±SEM. *p*-values were calculated by Student’s *t*-test or Mann–Whitney U test for **(A)** and **(B)**, or by ANOVA or Kruskal-Wallis for **(C)**, as appropriate, *p < 0.05, **p < 0.01.

We first quantified lymphocyte counts, as well as the numbers and percentages of cDCs and their subsets (CD1c^+^ and CD141^+^ cDCs), and pDCs in the PB of individuals with MDD and HC using a flow cytometry panel designed to distinguish DC subsets and their cytokine-producing populations. The two groups showed comparable lymphocyte counts. cDC counts were also equivalent, whereas pDCs were reduced in individuals with MDD compared to HC (Figure 1A). Frequencies of cDCs and their subsets (CD1c^+^ and CD141^+^ cDCs), as well as pDCs were similar across groups (Figure 1B). However, stratification by depression severity using the HAM-D17 scale revealed increased frequencies of CD11c^+^ cDCs in individuals with severe MDD (HAM-D17 ≥ 17) compared to those with mild symptoms (HAM-D17 9–16) or non-depressed participants (HAM-D17 < 9; Figure 1C). Moreover, cell counts showed a reduction of circulating pDCs in severe MDD compared to HC (Supplementary Figure 2).

### 3.3 Enhanced IL-23^+^cDCs frequencies in PB in MDD compared to HC

**Figure 2:**
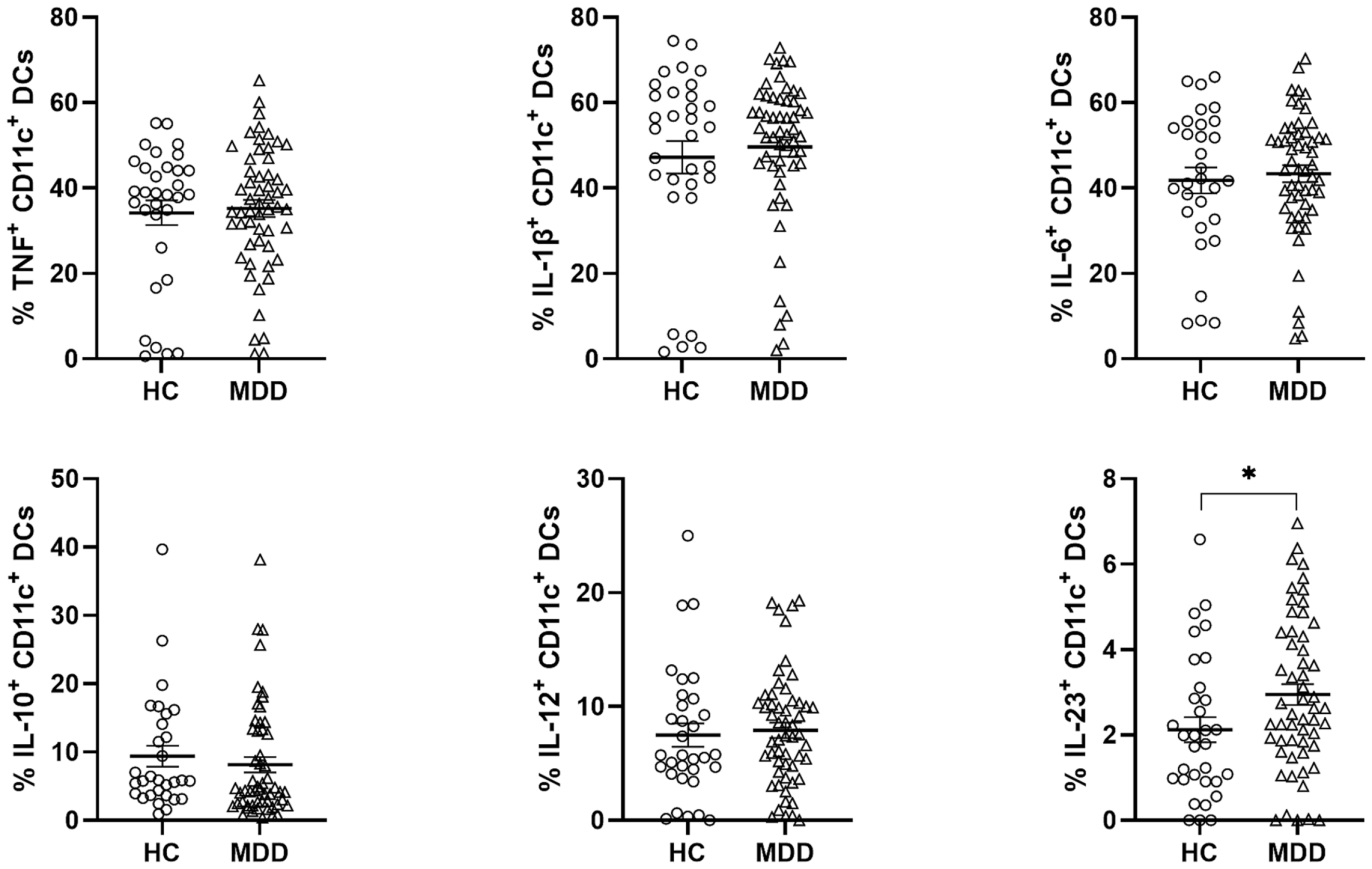
The frequency of circulating IL-23^+^ cDCs is elevated in patients with MDD. Graphs display proportions of DC subsets determined by flow cytometry in PB of cytokine producing cDCs in HC and patients with MDD. Error bars indicate mean ±SEM. *p*-values were calculated by Student’s *t*-test or Mann–Whitney U test, as appropriate, *p < 0.05.

Given the established involvement of pro- and anti-inflammatory cytokines in the pathophysiology of MDD (Dowlati et al., 2010), we next analyzed DC subsets producing TNF, IL-1β, IL-6, IL-10, IL-12, and IL-23 by intracellular staining, focusing on cDCs as the principal DC population responsible for production of these cytokines (Cabeza-Cabrerizo et al., 2021). Frequencies and absolute numbers of CD11c^+^ cDCs producing TNF, IL-1β, IL-6, IL-12, or IL-10 were comparable between individuals with MDD and HC. In contrast, IL-23^+^ cDC frequencies and counts were significantly higher in individuals with MDD compared to HC (Figure 2; Supplementary Figure 3A). Cytokine expression levels, assessed by mean fluorescence intensity (MFI), were similar between groups, indicating that while more IL-23^+^ cDCs were present in MDD, individual cells produced comparable amounts of IL-23 (Supplementary Figure 3B). Furthermore, no correlation was found between cytokine-producing cDC frequencies, counts, and BMI, excluding a confounding effect of BMI in depressed individuals (Supplementary Table 3). Together, these findings indicate that circulating IL-23^+^ cDCs are elevated in individuals with MDD compared to HC.

### 3.4 Differential frequencies of cytokine-producing DCs in females and males with MDD

**Figure 3:**
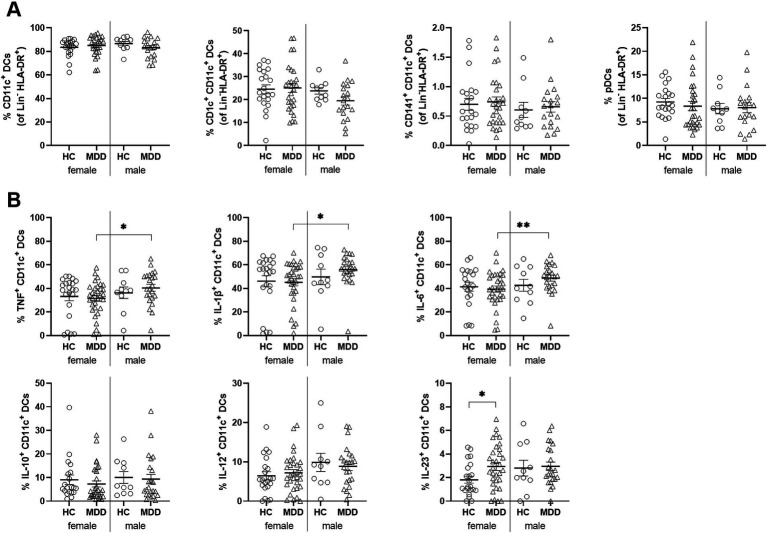
Increase in IL-23^+^ cDC frequencies in female but not male patients with MDD. Frequencies of DC subsets **(A)** and cytokine producing cDCs **(B)** in HC and patients with MDD stratified for sex. Error bars indicate mean ± SEM. *p*-values were calculated by Student’s *t*-test or Mann–Whitney U test, as appropriate, *p < 0.05, **p < 0.01.

To assess the impact of sex on DC profiles in MDD, we stratified patients and HC by sex and analyzed DC frequencies and counts in each group. All groups were matched for age, BMI, and smoking status. Male and female patients were additionally matched for depression severity based on BDI-II, HAM-D17, and IDS-C scores (Supplementary Table 1). cDC counts were comparable across groups, whereas pDCs were reduced only in female patients (Supplementary Figure 4). DC subset frequencies were otherwise similar (Figure 3A). As shown in Figure 3B, IL-10^+^ and IL-12^+^ cDC frequencies were comparable among all groups. In contrast, IL-23^+^ cDCs were elevated in females with MDD compared to sex-matched controls but unchanged in males. Conversely, depressed males displayed significantly higher proportions of TNF^+^, IL-1β^+^, and IL-6^+^ cDCs than females with MDD (Figure 3B). These findings indicate distinct, sex-specific cytokine-producing cDC profiles in MDD.

### 3.5 IL-23^+^ cDC frequencies are associated with MDD symptoms in females, but not males

**Figure 4:**
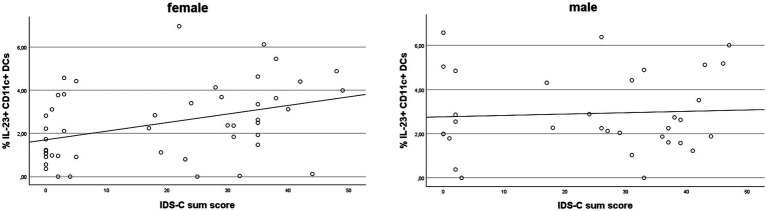
Blood frequencies of IL-23^+^ cDCs correlate with the IDS-C sum score in females but not males. Graphs show associations between IL-23 producing cDCs and the IDS-C sum score in female (left) or male (right) participants. Data were generated based on flow cytometry measurements, the IDS-C questionnaire, and subsequent correlational analyses between immune parameters and items related to MDD.

We next examined associations between cytokine-producing cDCs and depressive symptoms in females and males using both self-rated and clinician-rated depression scales (Table 2; Supplementary Table 4). Among all cytokine-producing DC subsets, only IL-23^+^ cDCs showed significant positive correlations with nearly all domains and the total score of the BDI-II in females after correction for multiple comparisons. These associations encompassed core depressive symptoms, including sadness, loss of pleasure, and reduced energy, as well as overall symptom severity. No significant correlations were observed in males (Supplementary Table 4). Using the IDS-C, IL-23^+^ cDCs again correlated significantly (after correction) with multiple symptom domains and the total score in females, but not in males (Table 2; Figure 4). Collectively, these results indicate a sex-specific relationship between IL-23^+^ cDCs and depressive symptomatology and severity in females, but not males, as reflected by both the BDI-II and IDS-C scales.

**Table 2:** Spearman rho correlation coefficients of cytokine-producing CD11c^+^ cDCs in females and males with MDD and HC with IDS-C items.

IDS-C items	% CD11c^+^ cDCs											
	TNF^+^		IL-1β^+^		IL-6^+^		IL-10^+^		IL-12^+^		IL-23^+^	
	Female	Male	Female	Male	Female	Male	Female	Male	Female	Male	Female	Male
Sleep onset insomnia	−0.015	0.194	−0.108	0.338	−0.111	0.280	0.007	−0.067	0.127	0.080	0.200	−0.067
Mid-nocturnal insomnia	−0.038	−0.036	−0.019	0.051	0.062	0.037	−0.174	−0.154	0.071	−0.269	0.369**	−0.290
Early morning insomnia	−0.190	−0.045	−0.151	0.020	−0.223	0.030	0.008	−0.146	−0.002	−0.215	0.120	−0.322
Hypersomnia	0.015	0.046	−0.007	−0.039	−0.034	−0.035	−0.062	−0.053	0.068	−0.253	0.066	0.040
Sad mood	−0.201	0.111	−0.171	0.173	−0.119	0.138	−0.187	−0.084	0.098	−0.044	0.351*	0.096
Irritable mood	−0.075	−0.050	−0.038	0.133	0.009	−0.045	−0.032	0.150	0.009	0.311	0.279*	0.310
Anxious mood	−0.221	0.226	−0.122	0.116	−0.157	0.310	−0.287*	−0.049	0.111	0.239	0.365**	0.245
Reactivity of mood	−0.169	0.094	−0.023	0.189	−0.102	0.161	−0.094	0.017	0.182	−0.014	0.457**	0.096
Mood variation	−0.073	0.076	−0.013	0.176	−0.031	0.041	−0.313*	0.096	0.083	0.071	0.197	0.140
Mood var.: time	−0.394	0.395	−0.578**	−0.228	−0.444*	−0.193	−0.372	−0.183	−0.133	−0.238	0.077	−0.095
Mood var.: environment	−0.238	−0.220	−0.293	0.019	−0.246	−0.077	−0.275	0.268	−0.367	−0.029	−0.210	−0.038
Quality of mood	−0.092	0.128	−0.016	0.072	0.028	0.228	−0.057	−0.281	−0.021	−0.066	0.230	0.067
Decreased appetite	−0.061	0.303	−0.090	0.421*	−0.042	0.347*	−0.281	0.145	−0.064	0.114	0.114	0.087
Increased appetite	−0.221	−0.024	−0.151	−0.272	−0.184	−0.191	−0.053	−0.236	−0.039	−0.267	−0.162	−0.028
Weight decrease	0.014	0.228	−0.106	0.247	−0.069	0.236	−0.300*	0.192	−0.143	0.050	−0.160	0.053
Weight increase	−0.259	−0.126	0.023	−0.379*	−0.179	−0.367*	0.166	−0.292	0.021	−0.282	0.109	−0.144
Concentration/decision making	−0.235	−0.029	−0.108	0.042	−0.140	0.001	−0.088	−0.091	0.147	−0.003	0.440**	−0.029
Outlook on self	−0.239	0.161	−0.128	0.033	−0.175	0.203	0.030	0.007	0.104	−0.041	0.360**	0.062
Outlook on future	−0.139	0.359*	−0.059	0.238	−0.064	0.304	−0.286*	0.086	0.117	0.148	0.252	0.244
Suicidal ideation	−0.073	−0.038	−0.161	0.082	−0.096	0.024	−0.195	−0.031	−0.035	−0.042	−0.015	0.033
Involvement	−0.140	0.155	−0.067	0.256	−0.099	0.266	−0.188	−0.052	0.128	0.015	0.375**	0.058
Energy/fatiguability	−0.255	0.092	−0.140	0.079	−0.172	0.109	−0.012	−0.276	0.137	−0.235	0.442**	−0.009
Pleasure/enjoyment	−0.144	0.081	−0.103	0.186	−0.101	0.061	−0.294*	−0.022	0.204	−0.229	0.294*	−0.064
Sexual interest	−0.188	−0.143	−0.114	0.063	−0.128	0.167	−0.214	−0.305	0.089	−0.166	0.289*	−0.038
Psychomotor. slowing	0.055	0.273	0.116	0.069	0.192	0.292	−0.003	−0.265	0.073	−0.208	0.113	0.015
Psychomotor. agitation	−0.199	−0.056	−0.149	−0.022	−0.134	0.057	0.059	−0.054	0.031	−0.173	0.070	−0.114
Somatic complaints	−0.164	0.089	−0.061	0.119	−0.235	0.173	−0.142	−0.177	0.178	−0.259	0.321*	0.000
Sympathetic arousal	−0.143	0.191	0.044	0.085	−0.075	0.245	−0.073	−0.144	0.268	0.013	0.504**	−0.101
Panic/phobic symptoms	0.059	0.067	0.077	−0.080	−0.004	0.027	−0.166	0.253	0.130	0.147	0.101	0.087
Gastrointestinal	−0.129	0.353*	−0.007	0.117	−0.057	0.219	−0.224	−0.116	0.033	0.115	0.162	−0.053
Interpersonal sensitivity	−0.223	0.200	−0.193	0.173	−0.137	0.107	0.038	0.387*	0.054	0.173	0.249	0.153
Leaden paralysis/physical energy	−0.347*	0.056	−0.276*	0.175	−0.331*	0.133	−0.071	0.011	0.073	−0.195	0.239	−0.016
IDS-C sum score	−0.296*	0.145	−0.154	0.127	−0.240	0.166	−0.195	−0.091	0.187	−0.144	0.429**	0.035

IDS-C, Inventory of Depressive Symptomatology–clinical interview. Underlined values show significant correlation coefficients after adjustment for multiple testing using Benjamini-Hochberg correction. *p < 0.05; **p < 0.01. Significant effects are in bold print.

### 3.6 Frequencies of cytokine producing cDCs predict affective/cognitive symptoms and severity of MDD in females but not males

We next performed multivariate linear regression (MLR) analysis to assess the predictive value of immune parameters (frequencies of cytokine-producing DCs) for a combined affective/cognitive symptom score of the IDS-C in female and male participants (Supplementary Table 5). IDS-C items were grouped according to affective/cognitive domains (Supplementary Table 6), and analyses were controlled for age, BMI, and smoking. The model did not reveal significant effects for male participants. In contrast, in females, IL-23^+^ cDCs, TNF^+^ cDCs, IL-10^+^ cDCs, age, and BMI significantly predicted the IDS-C affective/cognitive score (HC = 19, MDD = 25), explaining 47.1% of the variance [F (9, 34) = 3.360, *p* < 0.01, R^2^ = 0.471]. Additionally, the same model was used to predict the overall sum score of the IDS-C (HC = 18, MDD = 24), identifying the same significant factors for the female group and explaining 51.8% of the variance [F (9, 32) = 3.815, *p* < 0.01, R^2^ = 0.518] (Table 3). Again, the model for male participants did not reveal significance. These findings suggest that circulating cytokine-producing DC subsets, together with BMI and age, may serve as predictors of depressive symptoms and severity in females.

**Table 3:** Multivariate linear regression analysis of the IDS-C sum score in female and male participants.

Variable	B	SE	Beta	T	*p*-value
Female					
**IDS-C sum score (Constant)**	−32.105	25.918		−1.239	0.224
Age	0.526	0.196	0.410	2.680	**0.012**
BMI [kg/m^2^]	1.513	0.552	0.426	2.739	**0.010**
Smoking	−2.022	4.929	−0.056	−0.410	0.684
pDCs [%]	0.382	0.627	0.106	0.609	0.547
CD1c^+^ cDCs [%]	0.099	0.386	0.057	0.258	0.798
CD141^+^ cDCs [%]	−15.055	7.860	−0.410	−1.915	0.064
IL-23^+^ cDCs [%]	5.645	1.704	0.532	3.314	**0.002**
TNF^+^ cDCs [%]	−0.589	0.199	−0.504	−2.956	**0.006**
IL-10^+^ cDCs [%]	−0.699	0.295	−0.328	−2.366	**0.024**
IDS-C sum score: *F* = 3.815; df (9, 32); *p* < 0.01; R = 0.719; R^2^ = 0.518					
Male					
**IDS-C sum score (Constant)**	−18.646	39.922		−0.467	0.646
Age	−0.102	0.481	−0.044	−0.213	0.834
BMI [kg/m^2^]	2.106	1.183	0.442	1.781	0.092
Smoking	0.185	10.412	0.005	0.018	0.986
pDCs [%]	0.203	1.067	0.050	0.190	0.851
CD1c^+^ cDCs [%]	−1.243	0.672	−0.534	−1.850	0.081
CD141^+^ cDCs [%]	7.793	9.355	0.181	0.833	0.416
IL-23^+^ cDCs [%]	0.409	2.568	0.046	0.159	0.875
TNF^+^ cDCs [%]	0.212	0.306	0.186	0.695	0.496
IL-10^+^ cDCs [%]	−0.078	0.540	−0.043	−0.145	0.887
IDS-C sum score: *F* = 1.462; df (9, 18); *p* = 0.235; R = 0.650; R^2^ = 0.422					

Significant effects are in bold print.

### 3.7 Cluster analysis of cytokine-producing DCs in men and women with MDD

**Figure 5:**
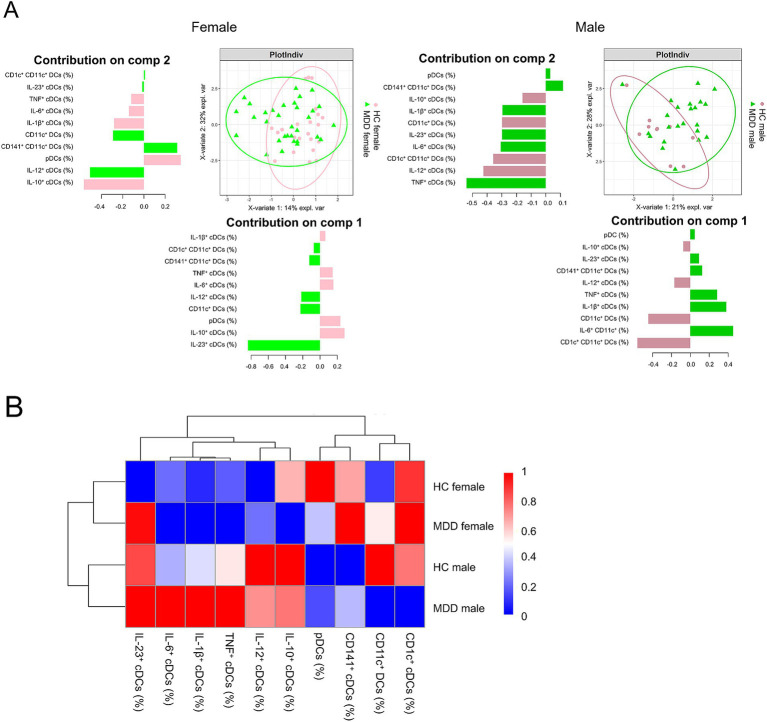
Sex-specific cellular immune profiles in MDD. Partial least squares discriminant analysis (PLS-DA) was performed to separate patients with MDD from the respective HC sex-matched group **(A)** based on cellular components. Mean frequencies of all obtained (cytokine producing) DC subsets were visualized in a hierarchical heatmap stratified for the sex and disease specific groups **(B)**.

Partial least squares discriminant analysis (PLS-DA) was performed to assess differences in cytokine-producing cDC subset frequencies between MDD patients and HC, separately for females and males (Figure 5A). In both analyses, a moderate separation between MDD and HC groups was observed, indicating distinct cDC subset profiles associated with MDD. The degree of discrimination was comparable between females and males. In females, group separation was primarily driven by IL-23^+^ cDCs on the first component and IL-10^+^ and IL-12^+^ cDCs on the second component. In males, CD1c^+^ cDCs and TNF^+^ cDCs contributed most strongly to the first and second components, respectively, identifying these subsets as principal discriminators of disease status in males. Next, a heatmap illustrating average DC subset frequencies across all groups was generated, with red indicating higher and blue lower cell percentages (Figure 5B). Hierarchical clustering revealed two main subclusters: female HC and MDD grouped together, as did male HC and MDD, indicating greater within-sex similarity. At a higher hierarchical level, both sex-based clusters merged, reflecting overall similarity across groups but distinct sex-specific profiles. Notably, the heatmap also shows that male MDD patients display the highest frequencies of TNF^+^, IL-1β^+^, and IL-6^+^ cDC subsets, suggesting a more proinflammatory DC profile in this group.

## 4 Discussion

In this exploratory study, we characterized circulating DC subsets and their cytokine-producing profiles in individuals with MDD compared with HC, with a particular focus on sex-specific immune alterations. Although overall DC subset frequencies were largely comparable between groups, several notable cellular patterns emerged. We observed a reduction of circulating pDC numbers, particularly in severe MDD, and a selective increase in cDC and IL-23^+^ cDC percentages in MDD. Moreover, we identified a distinct sex-specific DC profile in this study. Females with MDD exhibited elevated circulating IL-23^+^ cDCs that correlated with depressive symptom items and severity, whereas males displayed higher proportions of TNF^+^, IL-1β^+^, and IL-6^+^ cDCs without significant associations with symptom items and severity. These findings suggest a sex-dependent peripheral DC pattern in MDD, with women showing enhanced circulating IL-23^+^ DCs and men exhibiting a bias toward a more proinflammatory cDC cytokine profile.

pDCs can promote immunity via type I IFN production or regulate immune responses by inducing Treg cells. Depending on the context, these functions may confer host protection or contribute to pathology (Bauer et al., 2016). The observed reduction in pDCs is consistent with stress hormone-related suppression of type I IFN-producing pDCs in PB in humans (Lepelletier et al., 2010; Shodell et al., 2003; Shodell and Siegal, 2001). Human pDCs are highly glucocorticoid-sensitive and undergo glucocorticoid-induced apoptosis. Likewise, hyperinflammatory states (e.g., severe viral infection or critical illness) show lower circulating pDCs and dampened type I IFN activity despite elevated pro-inflammatory cytokines (Grimaldi et al., 2011; Hadjadj et al., 2020; Lu et al., 2025). It is thus possible that chronic stress in MDD may contribute to a contraction of the pDC/type I IFN compartment, as seen in stress-exposed human settings. In excluding a link between pDCs and depressive items and severity, our findings differ from a recent study showing that pDCs predicted improvement in hopelessness following 3 months of treatment of acute depression (Stolfi et al., 2025). Thus, pDC alterations may be state-dependent and modulated by inflammation or pharmacological treatment. The expansion of IL-23^+^ cDCs, on the other hand, represents a more specific immune profile in MDD. Since cytokine expression on a single cell level was equivalent in all groups, our finding reflects an increase in the proportion of IL-23 producing cDCs rather than enhanced cytokine production capacity on a single cell level. This selective increase of peripheral IL-23^+^ cDCs in depression points to an altered differentiation or activation of cDCs in the context of chronic stress and low-grade inflammation.

Sex-stratified analyses revealed that these immune alterations occurred primarily in depressed females. After controlling for age, BMI, smoking, and depression severity, we found that pDCs were reduced and IL-23^+^ cDCs were elevated in the PB only in female patients, compared to sex-matched controls. In contrast, males with MDD showed increased frequencies of TNF^+^, IL-1β^+^, and IL-6^+^ cDCs compared with female patients, but no difference in circulating IL-23^+^ cDCs. Cluster and discriminant analyses confirmed that DC profiles segregated primarily by sex. The lower IL-23^+^ cDC frequencies in healthy women compared with the other groups suggest that depression induces a shift from a homeostatic state comprising a low IL-23 producing DC profile to a more IL-23-skewed phenotype in cDCs in MDD that is specific to females.

Several underlying immune mechanisms may explain altered IL-23^+^ cDC frequencies in depressed females compared to sex-matched controls, such as increased expansion, enhanced cell survival and/or altered redistribution of functional DC subsets. Accordingly, sex hormones profoundly influence DC differentiation and responsiveness. Estrogens enhance NF-κB-dependent cytokine transcription and TLR signaling, whereas progesterone and androgens have been shown to suppress DC activation (Butts et al., 2007; Laffont et al., 2014; Mackern-Oberti et al., 2017). Under chronic stress and elevated glucocorticoids, as observed in MDD, this can lead to a paradoxical immune state where some DC functions are suppressed while others, such as IL-23 production, are amplified. Depressed women may therefore exhibit an exaggerated IL-23 response due to a convergence of estrogen signaling, glucocorticoid exposure, and stress-induced inflammatory cues. Furthermore, the IL-23/Th17 axis is biased toward females in autoimmune disorders such as multiple sclerosis and atopic dermatitis, both of which involve DC-derived IL-23 as a key pathogenic driver (Li et al., 2019; Vaknin-Dembinsky et al., 2006). Activation of IL-23/Th17 axis as well as elevated IL-23 blood levels have been described in depression (Daray et al., 2024; Moulton et al., 2025; Galecka et al., 2021), albeit the cellular source of IL-23 has remained unclear. Our data suggest that circulating cDCs are a cellular source of this cytokine. The interaction between DCs and DAMPs may also contribute to this effect. Chronic psychological stress and neuroinflammation elevate DAMPs such as high-mobility group box 1 (HMGB1), S100 proteins, and heat shock proteins, which signal through TLR2, TLR4, or RAGE on cDCs (Ma et al., 2024). Although the role of DAMPs in MDD is still being elucidated, growing evidence supports their involvement in its pathophysiology (Franklin et al., 2018; Peng et al., 2025; Serna-Rodriguez et al., 2022). These signals, especially when combined with cytokines such as GM-CSF, can induce IL-23 expression in DCs (Poppensieker et al., 2012). Our data are consistent with the idea that in depressed women, cDCs are more readily activated by DAMP-mediated pathways, whereas in healthy women, these cells remain in a more quiescent state. This hypothesis is supported by our previous work demonstrating that GM-CSF and CCR4 signaling promote IL-23 production in DCs in a murine model of CNS autoimmunity (Poppensieker et al., 2012; Ruland et al., 2017; Scheu et al., 2017). Given that some, though not all, studies have reported elevated GM-CSF levels in MDD (Annam et al., 2024; Hemmati et al., 2019), this pathway represents a plausible mechanism. Also, osteoprotegerin (OPG), a soluble member of the tumor necrosis factor receptor superfamily, has been shown to affect DC survival and cytokine production. LPS treatment of murine BM-derived OPG knock out DCs resulted in increased survival and elevated production of cytokines (TNF, IL-12, IL-23) of these cells compared to wildtype DCs, while adding OPG to the culture reduced their survival and cytokine production (Chino et al., 2009). Albeit, elevated OPG levels related to calcification and inflammation have been associated with severe mental disorders (Hope et al., 2010), postmenopausal women with MDD had decreased OPG levels compared to HC (Atteritano et al., 2013). Considering the mean age of 55 years in the female study group presented here, and the fact that around 55% of the MDD patients were either undergoing menopause or had reached the postmenopausal phase, it is possible that reduced OPG levels in female patients may contribute to the observed increased frequency of IL-23^+^ cDCs. Taken together, these findings suggest that the increase in IL-23^+^ cDCs in women with MDD reflects a biologically specific consequence of chronic stress, sex hormone modulation, and innate immune receptor activation.

The correlation analyses further strengthen the clinical relevance of this immune phenotype. IL-23^+^ cDC frequencies were positively associated with multiple depressive symptom domains and overall severity scores on both the BDI-II and IDS-C scales in females, whereas no significant associations were seen in males. Multivariate modeling further revealed that IL-23^+^, TNF^+^, and IL-10^+^ cDCs, together with BMI and age, predicted over 51% of the variance in MDD severity, as measured by the IDS-C sum score, in women, but not in men. This sex-specific linkage between IL-23^+^ cDCs and MDD severity suggests that these cells may not merely be markers but functional contributors to mood dysregulation. IL-23 and its downstream effector, IL-17, can influence the brain through microglial activation, increased blood–brain barrier permeability, and altered monoaminergic signaling, all mechanisms implicated in major depression (Gaffen et al., 2014). The pattern observed here suggests that in women with MDD, IL-23^+^ cDCs may reflect or promote a Th17-driven inflammatory milieu linked to affective dysregulation. The established association between Th17 activation and depressive symptom severity (Moulton et al., 2025; Beurel et al., 2013; Beurel and Lowell, 2018; Kim et al., 2021) further supports this interpretation. Moreover, the increase in CD11c^+^ DCs observed in severe MDD may influence chemokine signaling relevant to disease mechanisms. Consistent with this, we recently reported elevated plasma CCL17 levels, a chemokine primarily secreted by cDCs, in individuals with MDD (Freff et al., 2022). Peripheral blood IL-23^+^ cDCs may therefore reflect central immune processes driving depressive symptomatology in women or rather promote peripheral chronic inflammation with detrimental effects in MDD.

Our results have several translational implications. They add cytokine-producing DC phenotypes to the expanding list of immune alterations implicated in MDD. They also underscore that sex is not simply a covariate but a key biological stratifier. From a biomarker perspective, IL-23^+^ cDC frequencies could help define inflammatory subtypes of depression, potentially guiding immune-targeted interventions. Also from a public health perspective, implementing IL-23^+^ cDC screening could support assessment of severity of depression in females in the future. The findings also align with the concept of immuno-metabolic depression, a subtype characterized by low-grade inflammation and metabolic dysregulation with obesity, dyslipidaemia, and insulin resistance. This recently proposed concept of immuno-metabolic depression affects about 20–30% of patients (Penninx et al., 2025). Increased body weight in immuno-metabolic depression may influence IL-12 and IL-23 production in DCs; indeed, Sumarac-Dumanovic et al. reported upregulation of the IL-23/IL-17 axis in obese women, while weight loss reduced leptin and inflammatory cytokines, particularly IL-23 (Landgren et al., 2023; Sumarac-Dumanovic et al., 2009). Although IL-23^+^ cDCs did not correlate with BMI in our cohort, increased body weight and metabolic stress could amplify this inflammatory pathway, particularly in women, providing a link between metabolic state and immune activation in depression.

In this study, we focused on patients with MDD, however, other psychiatric disorders such as bipolar disorder (BD) and schizophrenia, are also frequently associated with immune alterations. A meta-analysis has shown that these mental disorders share common immune features, including altered peripheral blood cytokine profiles. In particular, schizophrenia and BD have been associated with elevated peripheral IL-23 blood levels (Debnath and Berk, 2017; Li et al., 2015), suggesting that DCs may contribute to immune dysregulation not only in MDD but also in other psychiatric disorders.

This study has several limitations. The sample size was moderate, and its cross-sectional design limits causal inference. Genetic risk factors for the development of MDD, such as *SLC6A4, BDNF,* and *CACNA1c*, the ladder coding for the pore-forming α1C subunit of the L-type calcium channel (LTCC) Ca_V_1.2, were not available for this study group. This is relevant because expression of Ca_V_1.2 channels by DCs has been shown to regulate, e.g., cytokine production and MHC-class II expression (Boller et al., 2025). Moreover, variants in *SLC6A4* and *BDNF* have been associated with altered IL-6 and BDNF levels in MDD, highlighting the potential contribution of genetic risk factors to immune alterations in depression (Colle et al., 2017; Su et al., 2009). In addition, circulating DCs were analyzed rather than tissue-resident or CNS-infiltrating cells, limiting conclusions about central mechanisms. Pharmacological treatments varied across participants, and potential drug effects on DC function cannot be fully excluded. Moreover, although cluster analyses indicated distinct sex-specific immune profiles, overall separation between MDD and HC was moderate, suggesting that DC alterations represent one component within a broader immune landscape in depression.

Future research should replicate these findings in larger, medication-free cohorts and incorporate longitudinal sampling to examine whether IL-23^+^ cDC numbers fluctuate with symptom changes or treatment response. In addition, our study highlights the importance of investigating DCs in the context of other mental disorders, both as potential biomarkers and potential future treatment targets. It further underscores the need for sex-stratified research in this field. Ultimately, prospective studies in larger cohorts within translational frameworks, such as the DFG funded Collaborative Research Centre 393 (SFB/TRR 393), could identify immune and metabolic predictors of illness trajectories and inform the development of sex-specific immunomodulatory strategies for affective disorders (Kircher et al., 2025).

In conclusion, our findings demonstrate that cytokine-producing DC subsets are altered in MDD in a sex-specific manner. These results identify IL-23-producing DCs as a potential immunological bridge between female sex, immune dysregulation, and depressive symptomatology, supporting the notion that sex-tailored immunomodulatory approaches may hold promise in the treatment of depression.

## Data Availability

The raw data supporting the conclusions of this article will be made available by the authors, without undue reservation.
