# Supplementary material for: Distinct cytokine-producing dendritic cell profiles in females and males with major depressive disorder
Source: Front Cell Neurosci. 2026 Feb 9;20:1753241. doi: 10.3389/fncel.2026.1753241 (PMC12926495; doi:10.3389/fncel.2026.1753241)
Supplement: Supplementary file 1 [file Supplementary_file_1.docx]

***Supplementary Material***





Supplementary Figure 1: Overview of the gating strategy for DC subsets. (A) Representative dot plots of a female MDD patient and sex-matched HC show the gating strategy for DCs and their subsets in PB as determined by flow cytometry. (B) Representative dot plots of cytokine producing cDCs in PB.


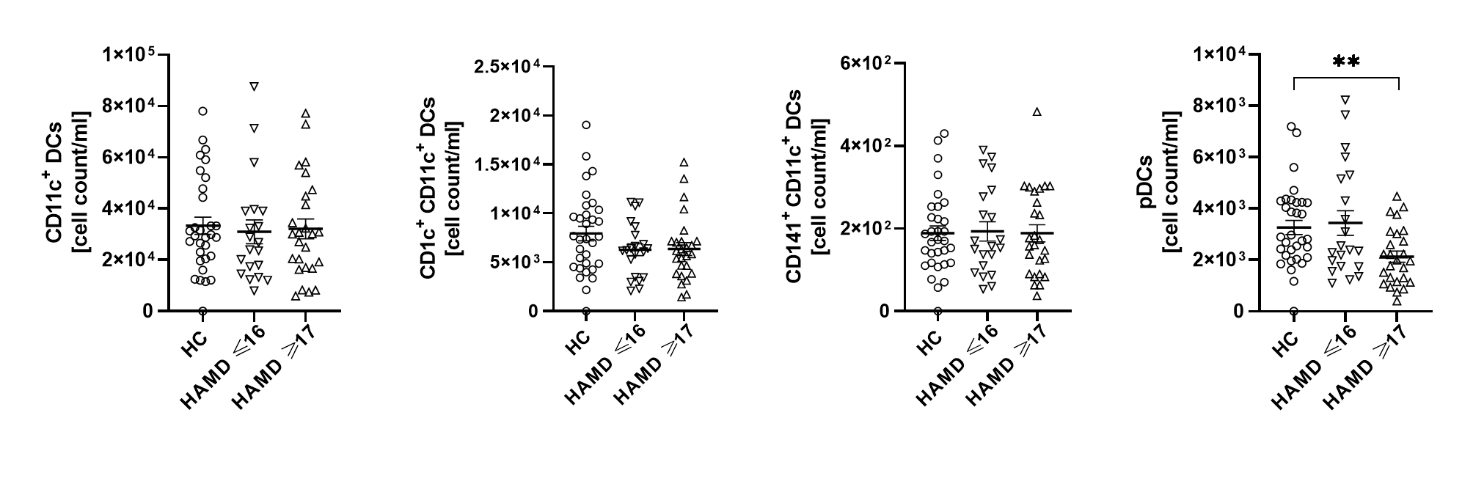
Supplementary Figure 2: Cell count/ml of DC subsets in MDD severity. Diagrams show mean values ±SEM of cell count/ml in participants stratified for MDD severity measured by the Hamilton Rating Scale for Depression (HAM-D17) into HC, mildly depressed (HAMD ≤16) and moderate to severely depressed (HAMD ≥17) patients. *p*-values were calculated by ANOVA or Kruskal-Wallis, as appropriate, ***p* < 0.01


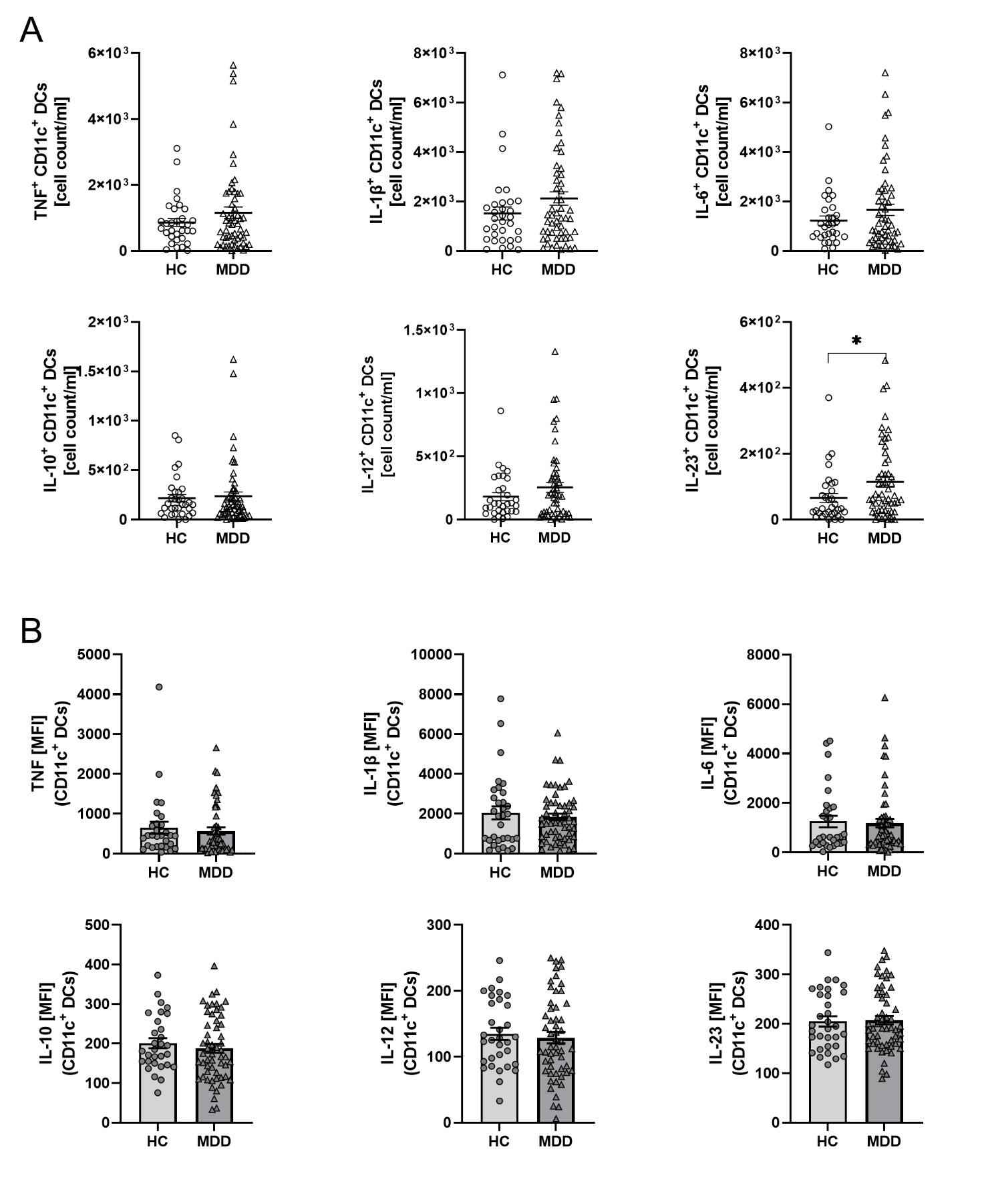
Supplementary Figure 3: Cell count/ml of cytokine producing cDCs and cytokine expression levels of DC subsets in the PB of HC and patients with MDD. (A) Diagram shows mean ±SEM of numbers of DC subsets in PB as determined by flow cytometry. (B) Graphs display median fluorescent intensity (MFI) of cytokine expression in cDCs of HC and MDD. *p*-values were calculated by Student’s *t*-test or Mann-Whitney U test, as appropriate, **p* < 0.05

Supplementary Figure 4: Graphs show mean cell count/ml ±SEM of CD11c^+^ cDCs and pDCs in HC and patients with MDD
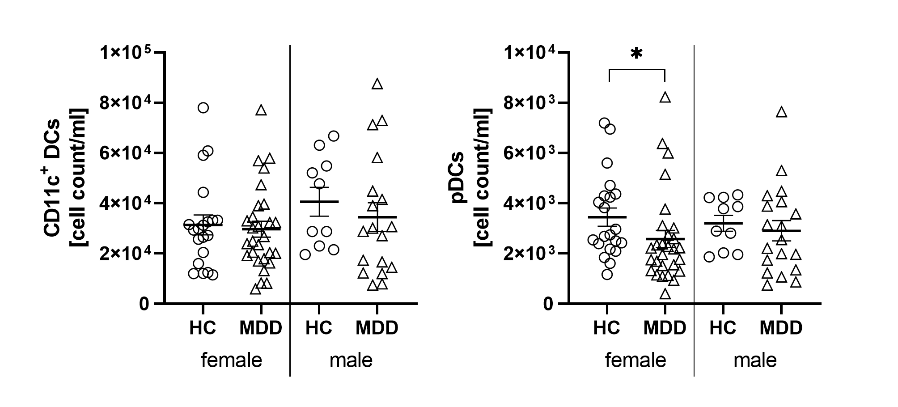
 stratified for sex. *p*-values were calculated by Student’s *t*-test or Mann-Whitney U test, as appropriate, **p* < 0.05

Supplementary Table 1: Demographics and clinical characteristics of the study sample stratified into female and male participants

|  | female | | |  | male | | |  | female vs. male | |
| --- | --- | --- | --- | --- | --- | --- | --- | --- | --- | --- |
|  | HC (*n* = 22) | MDD (*n* = 32) | *p*-value |  | HC (*n* = 10) | MDD (*n* = 23) | *p*-value |  | in HC *p*-value | in MDD *p*-value |
| Age ± SD | 52.32 ± 15.41 | 54.91 ± 12.74 | .504 |  | 57.20 ± 5.63 | 55.04 ± 8.29 | .460 |  | .202 | .962 |
| Smoking | 5 | 11 | .357 |  | 2 | 5 | 1.000 |  | 1.000 | .357 |
| BMI ± SD | 24.85 ± 4.79 | 26.41 ± 4.18 | .085 |  | 25.51 ± 2.97 | 28.04 ± 4.09 | .088 |  | .366 | .159 |
| HAM-D17 ± SD | 0.55 ± 0.91 | 17.53 ± 4.50 | **< .001***** |  | 0.40 ± 0.52 | 16.70 ± 4.11 | **< .001***** |  | .961 | .484 |
| IDS-C ± SD | 1.48 ± 1.75 | 32.37 ± 8.56 | **< .001***** |  | 1.20 ± 1.14 | 34.09 ± 8.43 | **< .001***** |  | .876 | .480 |
| BDI-II ± SD | 2.68 ± 2.82 | 28.77 ±11.78 | **< .001***** |  | 2.20 ± 2.30 | 28.30 ± 8.16 | **< .001***** |  | .679 | .876 |

HC: healthy controls; MDD: major depressive disorder; SD: standard deviation; BMI: body mass index; HAM-D17: Hamilton Rating Scale for Depression (17 questions); IDS-C: Inventory of Depressive Symptomatology – clinical interview; BDI-II: Beck Depression Inventory-II. Significant effects are in bold print. ****p* < .001.

Supplementary Table 2: Medical treatment of patients with MDD

| Medication | MDD | |
| --- | --- | --- |
|  | *n* (female/male) | *%* (female/male) |
| **Antidepressants** |  |  |
| Tricyclic antidepressants | 2/2 | 6/9 |
| Tetracyclic antidepressants | 16/11 | 50/48 |
| Agomelatine | 8/4 | 25/17 |
| SSRI | 6/4 | 19/17 |
| NARI | 0/0 | 0/0 |
| SSNRI | 16/15 | 50/65 |
| NDRI | 1/2 | 3/9 |
| MAOI | 3/0 | 9/0 |
| **Antipsychotics** |  |  |
| Atypical | 19/13 | 59/57 |
| Typical | 11/4 | 34/17 |
| **Mood stabilizer (Lithium)** | 1/3 | 3/13 |
| **Benzodiazepines** | 11/8 | 34/35 |
| **Anxiolytics** |  |  |
| Z-drugs | 16/5 | 50/22 |
| Anticonvulsive | 4/2 | 13/9 |
| **Others** | 2/4 | 6/17 |

Supplementary Table 3: Correlation between PB cell frequencies, count/ml, and BMI

|  | | | **% Lin^-^ DCs** | | | | | | |  | | | **% CD11c^+^ DCs** | | | | | | |  |
| --- | --- | --- | --- | --- | --- | --- | --- | --- | --- | --- | --- | --- | --- | --- | --- | --- | --- | --- | --- | --- |
|  | | CD11c^+^ | | | CD1c^+^ | | CD141^+^ | | pDCs | | |  | | TNF^+^ | IL-1β^+^ | IL-6^+^ | IL-10^+^ | IL-12^+^ | IL-23^+^ | |
| BMI [kg/m^2^] | | -0.030 | | | -0.023 | | 0.134 | | 0.032 | | |  | | -0.191 | 0.095 | -0.013 | 0.066 | 0.086 | 0.107 | |
| **Cell count/ml** | | | | | | | | | | | | | | | | | | | |  |
|  | CD11c^+^ | | | CD1c^+^ | | CD141^+^ | | pDCs | | |  | | | TNF^+^ | IL-1β^+^ | IL-6^+^ | IL-10^+^ | IL-12^+^ | IL-23^+^ | |
| BMI [kg/m^2^] | -0.113 | | | -0.172 | | -0.011 | | -0.167 | | |  | | | -0.033 | 0.134 | 0.076 | 0.103 | 0.091 | 0.177 | |

BMI: Body Mass Index

Supplementary Table 4: Spearman Rho correlation coefficients of cytokine-producing CD11c^+^ cDCs in females and males with MDD and HC

| BDI-II Items | % CD11c^+^ cDCs | | | | | | | | | | | |
| --- | --- | --- | --- | --- | --- | --- | --- | --- | --- | --- | --- | --- |
|  | TNF^+^ | | IL-1β^+^ | | IL-6^+^ | | IL-10^+^ | | IL-12^+^ | | IL-23^+^ | |
|  | female | male | female | male | female | male | female | male | female | male | female | male |
| Sadness | -0.160 | 0.002 | -0.135 | 0.082 | -0.153 | -0.017 | -0.159 | -0.317 | 0.113 | -0.167 | **.354**** | -0.104 |
| Pessimism | -0.185 | 0.178 | -0.071 | 0.274 | -0.066 | 0.212 | -0.014 | -0.084 | 0.119 | -0.096 | **.376**** | -0.078 |
| Past failure | -0.180 | 0.166 | -0.098 | 0.343 | -0.203 | 0.209 | -0.146 | 0.156 | 0.079 | 0.090 | **.281*** | 0.005 |
| Loss of pleasure | -0.161 | 0.005 | -0.123 | 0.137 | -0.169 | 0.054 | -0.127 | -0.242 | 0.064 | -0.26 | **.331*** | -0.256 |
| Guilty feelings | -0.111 | 0.259 | -0.055 | **.420*** | -0.115 | 0.280 | -0.009 | 0.176 | 0.25 | 0.15 | **.511**** | 0.099 |
| Punishment feelings | **-.280*** | 0.074 | -0.262 | 0.151 | **-.302*** | 0.147 | -0.262 | 0.073 | -0.045 | 0.153 | 0.191 | -0.063 |
| Self-dislike | -0.226 | 0.062 | -0.107 | 0.175 | -0.220 | 0.075 | -0.181 | 0.027 | 0.005 | -0.061 | **.370**** | -0.001 |
| Self-criticalness | -0.195 | 0.071 | -0.166 | 0.069 | -0.178 | 0.079 | -0.020 | -0.167 | 0.184 | **-.363*** | **.424**** | -0.254 |
| Suicidal thoughts or wishes | **-.358**** | -0.156 | -0.249 | 0.084 | **-.348*** | 0.108 | -0.232 | -0.140 | -0.017 | -0.084 | 0.234 | -0.176 |
| Crying | -0.157 | -0.028 | 0.045 | 0.113 | -0.099 | -0.013 | -0.026 | -0.171 | 0.223 | 0.03 | **.461**** | 0.033 |
| Agitation | -0.268 | 0.293 | -0.189 | 0.327 | -0.208 | 0.336 | -0.099 | 0.131 | 0.057 | 0.153 | **.389**** | 0.214 |
| Loss of interest | -0.234 | -0.073 | -0.102 | 0.047 | -0.167 | -0.063 | -0.242 | -0.354 | 0.154 | -0.115 | **.371**** | -0.105 |
| Indecisiveness | -0.133 | -0.076 | -0.009 | 0.031 | -0.049 | -0.043 | -0.123 | -0.159 | 0.194 | -0.153 | **.379**** | -0.108 |
| Worthlessness | -0.093 | 0.089 | -0.028 | -0.029 | -0.080 | 0.007 | -0.096 | -0.178 | 0.149 | -0.235 | **.330*** | -0.109 |
| Loss of energy | -0.218 | -0.120 | -0.087 | -0.060 | -0.211 | -0.079 | 0.010 | **-.383*** | 0.131 | -0.206 | **.375**** | -0.162 |
| Changes in sleeping pattern | -0.156 | 0.046 | -0.090 | 0.212 | -0.069 | 0.119 | -0.071 | -0.159 | 0.208 | -0.079 | **.407**** | 0.001 |
| Irritability | -0.182 | 0.094 | -0.164 | 0.234 | -0.118 | 0.123 | 0.008 | 0.023 | 0.012 | 0.097 | **.383**** | 0.099 |
| Changes in appetite | **-.316*** | **.371*** | **-.335*** | 0.246 | **-.291*** | 0.210 | -0.138 | 0.087 | -0.116 | 0.06 | 0.136 | 0.215 |
| Concentration difficulty | -0.256 | -0.073 | -0.155 | 0.106 | -0.229 | 0.003 | -0.138 | -0.179 | 0.084 | -0.142 | **.380**** | -0.069 |
| Tiredness or fatigue | -0.127 | -0.097 | -0.087 | -0.069 | -0.104 | -0.216 | -0.038 | -0.278 | 0.139 | **-.455*** | **.453**** | -0.261 |
| Loss of interest in sex | -0.213 | -0.218 | -0.105 | -0.010 | -0.120 | -0.012 | -0.056 | **-.422*** | -0.005 | -0.339 | 0.093 | -0.349 |
| BDI-II sum score | -0.238 | 0.041 | -0.169 | 0.129 | -0.200 | 0.021 | -0.095 | -0.204 | 0.12 | -0.177 | **.414**** | -0.138 |

BDI-II: Beck Depression Inventory-II. Underlined values show significant correlation coefficients after adjustment for multiple testing using Benjamini-Hochberg correction. **p* < .05; ***p* < .01

Supplementary Table 5: Multivariate linear regression analysis of a sub score of affective/cognitive IDS-C items in female or male participants

| FEMALE | | | | | |
| --- | --- | --- | --- | --- | --- |
| Variable | B | SE | Beta | T | *p*-value |
| **IDS-C affective/cognitive (Constant)** | -14.963 | 12.401 |  | -1.207 | 0.236 |
| Age | 0.235 | 0.094 | 0.373 | 2.501 | **0.017** |
| BMI [kg/m²] | 0.705 | 0.276 | 0.389 | 2.558 | **0.015** |
| Smoking | -0.243 | 2.514 | -0.013 | -0.097 | 0.924 |
| pDCs [%] | 0.206 | 0.325 | 0.112 | 0.632 | 0.531 |
| CD1c^+^ cDCs [%] | 0.071 | 0.198 | 0.082 | 0.360 | 0.721 |
| CD141^+^ cDCs [%] | -7.521 | 4.119 | -0.403 | -1.826 | 0.077 |
| IL-23^+^ cDCs [%] | 2.739 | 0.873 | 0.525 | 3.137 | **0.004** |
| TNF^+^ cDCs [%] | -0.281 | 0.104 | -0.469 | -2.707 | **0.011** |
| IL-10^+^ cDCs [%] | -0.338 | 0.152 | -0.308 | -2.217 | **0.033** |
| IDS-C affective/cognitive: F = 3.360; df (9, 34); *p* < 0.01; R = 0.686; R^2^ = 0.471 | | | | | |
|  |  |  |  |  |  |
| MALE | | | | | |
| Variable | B | SE | Beta | T | *p*-value |
| **IDS-C affective/cognitive (Constant)** | -5.420 | 21.517 |  | -0.252 | 0.804 |
| Age | -0.191 | 0.259 | -0.153 | -0.739 | 0.470 |
| BMI [kg/m²] | 1.129 | 0.637 | 0.436 | 1.771 | 0.094 |
| Smoking | -4.512 | 5.612 | -0.205 | -0.804 | 0.432 |
| pDCs [%] | 0.067 | 0.575 | 0.030 | 0.116 | 0.909 |
| CD1c^+^ cDCs [%] | -0.488 | 0.362 | -0.386 | -1.347 | 0.195 |
| CD141^+^ cDCs [%] | 2.185 | 5.042 | 0.093 | 0.433 | 0.670 |
| IL-23^+^ cDCs [%] | 1.214 | 1.384 | 0.249 | 0.877 | 0.392 |
| TNF^+^ cDCs [%] | 0.043 | 0.165 | 0.069 | 0.261 | 0.797 |
| IL-10^+^ cDCs [%] | 0.010 | 0.291 | 0.011 | 0.036 | 0.972 |
| IDS-C affective/cognitive: F = 1.519; df (9, 18); *p*=0.215; R = 0.657; R^2^ = 0.432 | | | | | |

Supplementary Table 6: IDS-C items included in the sub score of affective/cognitive items

| IDS-C item |  |  |  | IDS-C item |  |
| --- | --- | --- | --- | --- | --- |
| 5 | Mood (sad) |  |  | 15 | Concentration/decision making |
| 6 | Mood (irritable) |  |  | 16 | Outlook (self) |
| 7 | Mood (anxious) |  |  | 17 | Outlook (future) |
| 8 | Reactivity of mood |  |  | 18 | Suicidal ideation |
| 9 | Mood variation |  |  | 27 | Panic/phobic symptoms |
| 10 | Quality of mood |  |  | 29 | Interpersonal sensitivity |

IDS-C: Inventory of Depressive Symptomatology – clinical interview
